# Effects of Seaweed Polysaccharide (SP) and Seaweed Enzymatic Hydrolysate (SEH) on Growth Performance, Antioxidant Capacity, Immune Function, and Gut Microbiota in Muscovy Ducks

**DOI:** 10.3390/ani15203047

**Published:** 2025-10-20

**Authors:** Hong-Yan Wu, Xiao-Feng Lin, Chang-Sheng Fu, Yang Yang, Lei Wang, Hai-Yan Wu, Pan-Pan Guo, Deng-Feng Wang, Guang-Wen Yin

**Affiliations:** College of Animal Sciences, Fujian Agriculture and Forestry University, Fuzhou 350002, China

**Keywords:** seaweed polysaccharide (SP), seaweed enzymatic hydrolysate (SEH), feed additives, growth performance, antioxidant capacity, gut microbiome, Muscovy ducks

## Abstract

This experiment investigated the effects of seaweed polysaccharide (SP) and seaweed enzymatic hydrolysate (SEH) on the growth performance, serum biochemical indices, antioxidant capacity, and intestinal function of Muscovy ducks. A total of 240 healthy 1 day female Muscovy ducks (48.85 ± 0.45 g) were randomly divided into 3 treatment groups, with 4 replicates per group and 20 ducks per replicate. The control (CON) group received a basic diet supplemented with 20 mL/kg of water, the SP group received a basic diet supplemented with 20 mL/kg of SP, and the SEH group received a basic diet supplemented with 20 mL/kg of SEH. The experimental period lasted for 28 d. The results indicate that, compared to the CON group, the average daily feed intake (ADFI) and feed to gain (F/G) of the SP and SEH groups of ducks significantly decreased at 28 d (*p* < 0.05). In the SP group, serum levels of alanine aminotransferase (ALT) and aspartate aminotransferase (AST), as well as the concentrations of glucose (GLU), triglycerides (TG), total cholesterol (TCHO), low-density lipoprotein cholesterol (LDL-C), and high-density lipoprotein cholesterol (HDL-C), were significantly reduced (*p* < 0.05). In the SEH group, the activities of ALT and AST were also significantly lower (*p* < 0.05). Additionally, serum total antioxidant capacity (T-AOC) levels and superoxide dismutase (SOD) activity in the SEH group were significantly higher than those in the CON group (*p* < 0.05), while the malondialdehyde (MDA) content was significantly reduced (*p* < 0.05). Compared to the CON group, serum levels of immunoglobulin A (IgA), immunoglobulin G (IgG), interleukin-4 (IL-4), and interleukin-10 (IL-10) in the SP group were significantly increased (*p* < 0.05), whereas the levels of tumor necrosis factor-alpha (TNF-α), interleukin-1 beta (IL-1β), and interleukin-6 (IL-6) were significantly decreased (*p* < 0.05). In the SP and SEH groups, the villus height (VH) and the villus height to crypt depth (V/C) of the Muscovy ducks significantly increased (*p* < 0.05), while the crypt depth (CD) significantly decreased (*p* < 0.05). A significant increase in the abundance of *Barnesiella* was observed in the SP and SEH groups (*p* < 0.05), whereas the abundances of *UCG-005* and *Romboutsia* significantly decreased (*p* < 0.05). LEfSe analysis indicated that *g__Bacillus* and *g__Veillonella* were significantly abundant in the SP group (*p* < 0.05), while *g__Coriobacteriaceae_UCG_002* was significantly abundant in the SEH group (*p* < 0.05). In summary, the addition of SP and SEH to the feed can promote the healthy growth of ducks by improving intestinal morphology, regulating the structure of intestinal microbiota, enhancing antioxidant capacity and immune function, and optimizing metabolic indicators. This occurs while reducing feed intake and feed-to-weight ratio, and there is a certain specificity in their mechanisms of action.

## Simple Summary

This study investigated the effects of seaweed polysaccharide (SP) and seaweed enzymatic hydrolysate (SEH) on 240 one-day-old female Muscovy ducks over a 28-day period. The ducks were categorized into three groups: a control group (CON) that received a basic diet supplemented with 20 mL/kg of water, an SP group that received 20 mL/kg of SP, and an SEH group that received 20 mL/kg of SEH. The results indicated that both SP and SEH significantly reduced the average daily feed intake and feed-to-gain ratio of the ducks. Additionally, they improved intestinal morphology by increasing villus height and the villus height-to-crypt depth while decreasing crypt depth, and they also regulated intestinal microbiota. Furthermore, SEH enhanced serum antioxidant capacity, while SP improved immune function. Both treatments optimized metabolic indicators, thereby promoting healthy growth in ducks, although their mechanisms exhibited some specificity.

## 1. Introduction

In the livestock industry’s transition to efficiency and sustainability, how to enhance breeding efficiency while cutting antibiotics and other chemicals is critical for its sustainable development [1]. The Muscovy duck, as an important specialty waterfowl variety in southern China, is in high demand in the market due to its firm meat, unique flavor, and high nutritional value [2]. However, during its breeding process, it often faces problems such as poor intestinal health and low immunity, which restrict further improvement of production performance [3,4]. Therefore, the development of safe and efficient natural feed additives has become a crucial breakthrough for improving the current situation of Muscovy duck farming.

Marine algae, as one of the oldest photosynthetic organisms on Earth, have emerged as an ideal source of natural feed additives due to their rich bioactive components and renewable characteristics [5]. Among them, seaweed polysaccharide (SP) serve as the core functional component of algae, which have been demonstrated to possess various biological activities such as antioxidant, immune regulation, and improvement of gut microecology, showcasing promising application potential in livestock and poultry farming [6,7]. Moreover, seaweed enzymatic hydrolysates (SEH) are prepared through enzymatic hydrolysis technology. This technology not only retains the active substances of algae but also converts the macromolecular components in algae into smaller molecular fragments [8]. These smaller molecular fragments are more easily absorbed and utilized by animals, which further enhances the bioavailability of SEH and provides new insights for overcoming the application bottlenecks of natural extracts in waterfowl [9].

Currently, the application of SP in livestock and poultry farming has been extensively studied, with their positive effects such as promoting growth and enhancing disease resistance being thoroughly validated. However, research related to SEH is relatively scarce [10,11,12]. Notably, compared to common livestock and poultry such as chickens and pigs, systematic studies on SP and SEH concerning the Muscovy duck, are even more limited. Given the unique physiological and metabolic characteristics and breeding needs of Muscovy ducks, exploring the effects of SP and SEH on their growth performance, immune function, and intestinal health can not only enrich the application theory of marine bioactive substances in waterfowl breeding, but also provide scientific basis for promoting the development of green and healthy breeding models for Muscovy ducks.

## 2. Materials and Methods

### 2.1. Ethics Statement

The experimental procedures adhered to the guidelines for the care and use of experimental animals established by the National Research Commission. This study received approval from the Ethics Committee of the School of Animal Science and Technology at Fujian Agriculture and Forestry University, under reference number PZCASFAFU25059.

### 2.2. Experimental Design and Duck Management

In this experiment, 240 healthy, weight-consistent 1 d Changlong breed female Muscovy ducks (48.85 ± 0.45 g) were randomly divided into 3 treatment groups, with 4 replicates per group and 20 ducks per replicate. The control (CON) group received a basic diet (Table 1) supplemented with 20 mL/kg of water, the seaweed polysaccharide (SP) group received a basic diet supplemented with 20 mL/kg of SP, and the seaweed enzymatic hydrolysate (SEH) group received a basic diet supplemented with 20 mL/kg of SEH. The composite seaweed consists of 29% nori, 24% dulse, and 47% kelp. After treatment with cellulase and pectinase, followed by hydrolysis with neutral protease, aminopeptidase, and alginate lyase, it is processed into SEH. Measurements indicate that SEH contains soluble solids of 537.3 mg/g, which include total polysaccharides at 60.5 mg/g, reducing sugars at 38.4 mg/g, proteins at 2144.8 mg/g, and bound sulfate (SO_4_^2−^) at 5.6 mg/g. The total antioxidant capacity is 43.5 mmol FeSO_4_/g. The experimental period lasted for 28 d. The experiment was conducted at the Longhai Changnong experimental farm (Fujian, China), where the ducks were raised according to the routine management practices of the farm, with ad libitum feeding and watering.

### 2.3. Sample Collection and Parameter Determination

#### 2.3.1. Growth Performance

In the experiments conducted at 1 and 28 d, all ducks in each replicate group were weighed. At 28 d, the remaining feed was cleaned up, and the total feed intake was recorded. The average daily gain (ADG), average daily feed intake (ADFI), and feed to gain (F/G) of the ducks at 1–28 d were determined.

#### 2.3.2. Serum Indicators

On the 28th day, 1 duck from each replicate group, whose weight was close to the average, was selected. After a 12 h fasting period, blood was collected using a 5 mL vacuum tube without anticoagulant and was immediately placed on ice. After standing at an angle for 15 min, serum was collected by centrifugation at 2000× *g* for 10 min and stored at −20 °C for further analysis. Total protein (TP), albumin (ALB), alkaline phosphatase (ALP), alanine aminotransferase (ALT), aspartate aminotransferase (AST), glucose (GLU), triglyceride (TG), total cholesterol (TCHO), low-density lipoprotein cholesterol (LDL-C), and high-density lipoprotein cholesterol (HDL-C) in serum were measured using the Maikang Biological Automatic Analyzer (Maikang 1280, Foshan, China). The total antioxidant capacity (RXWB0465-96, T-AOC), superoxide dismutase (RXM700029D, SOD), catalase (RXM2D623656, CAT), glutathione peroxidase (RXM700028D, GSH-Px), malondialdehyde (JRXW622986, MDA), immunoglobulin A (RX700259D, IgA), immunoglobulin G (RX700258D, IgG), tumor necrosis factor-α (RX700248D, TNF-α), interleukin-1β (RX700140D, IL-1β), interleukin-6 (RX700270D, IL-6), transforming growth factor-β1 (RX2D646836, TGF-β1), interleukin-4 (RX700217D, IL-4), and interleukin-10 (RX700216D, IL-10) in serum were measured using kits from Quanzhou Ruixin Biological Technology Co., Ltd (Quanzhou, China).

#### 2.3.3. Intestinal Morphology

In the experiment conducted at 28 d, after the ducks were euthanized by decapitation, the abdominal cavity was opened under sterile conditions, and the intestinal contents were gently rinsed with pre-cooled saline. Approximately 1 cm of the jejunum was excised and fixed in 4% formalin for 24 h. The fixed tissue was then subjected to a dehydration process using a gradient of ethanol (70%, 80%, 90%, 95%, 100%), with each concentration for 2 h. After dehydration, the tissue was treated with xylene for 15 min to achieve transparency, followed by immersion in melted paraffin for embedding. The tissue was subsequently placed in embedding boxes, filled with melted paraffin, and allowed to cool and solidify into paraffin blocks. Using a microtome, the paraffin blocks were sliced into sections with a thickness of 4–6 μm, which were gently flattened in a water bath at approximately 40 °C. The sections were then collected on slides, air-dried at room temperature or dried in a 37 °C incubator. After deparaffinization with xylene and rehydration through a gradient of ethanol, the sections were stained using the conventional HE (Hematoxylin–Eosin) method and finally mounted with neutral resin. The villi height (VH), the crypts depth (CD), and the villi height to crypts depth (V/C) were measured. The VH and CD were measured utilizing Image Pro Plus 6.0 analysis software (Image Pro Plus 6.0).

#### 2.3.4. Gut Microbiota

At 28 d, the cecal contents of the Muscovy ducks were collected under sterile conditions and rapidly frozen in liquid nitrogen before being transferred to a −80 °C freezer for gut microbiome analysis. For genomic DNA isolation from cecal digesta samples, we utilized the EZNA stool DNA kit from Omega Biotek (Norcross, GA, USA). The concentration of the isolated DNA was measured using a NanoDrop 2000 spectrophotometer (Thermo Scientific, Waltham, MA, USA), and its quality was assessed through electrophoresis on 2% agarose gels. After DNA extraction and quality assessment, we amplified the V3-V4 region of the 16S rRNA gene, employing the extracted genomic DNA as a template along with specific primers 338F (5′barcodeACTCCTACGGGAGGCAGCAG3′) and 806R (5′GGACTACHVGGGTWTCTAAT3′). To purify the obtained PCR products, we utilized the AxyPrep DNA Gel Extraction Kit from Axygen Biosciences (Union City, CA, USA). The purified amplicons were combined in equal molar ratios in preparation for sequencing, which was conducted on the Illumina MiSeq platform. To ensure the integrity of the sequence data, we processed the raw Illumina fastq files with a quality-filtering step using Trimmomatic (version 3.29). Subsequently, operational taxonomic units (OTUs) were clustered at a similarity threshold of 97% using UPARSE (version 7.0), facilitating effective classification of the identified microbial communities.

### 2.4. Statistical Analysis

The General Linear Model (GLM) procedure in SAS 9.4 (SAS Institute Inc., Cary, NC, USA) was utilized for the statistical analysis of all data, with post hoc comparisons of treatment differences conducted using Tukey’s multiple range test. Results are expressed as the mean ± standard error of the mean (SEM). A *p* < 0.05 is considered to have a significant difference. For the analysis of gut microbiota in Muscovy ducks, the online services provided by the NovoMagic Cloud Platform (https://magic-plus.novogene.com) were employed.

## 3. Results

### 3.1. Growth Performance

The effects of SP and SEH on the growth performance of Muscovy ducks are presented in Table 2. Compared to the CON group, there were no significant differences in BW and ADG at 28 d between the SP and SEH groups (*p* > 0.05). Additionally, there were no significant differences in ADFI and F/G at 28 d between the SP and SEH groups (*p* > 0.05), both measures were lower than those observed in the CON group (*p* < 0.05).

### 3.2. Serum Biochemical Indicators

As shown in Table 3, compared to the CON group, the activities of ALT and AST in the serum of the Muscovy duck in the SP and SEH groups were significantly reduced (*p* < 0.05), with the SEH group exhibiting higher levels than the SP group (*p* < 0.05). Furthermore, when compared to the CON group, the levels of GLU, TC, TCHO, LDL-C, and HDL-C in the serum of the Muscovy duck in the SP group were significantly lower (*p* < 0.05), although there were no significant differences between the SEH and SP groups (*p* > 0.05).

### 3.3. Serum Antioxidant Capacity

Table 4 shows the effect of SP and SEH on the antioxidant capacity of Muscovy ducks serum. Compared to the CON group, the T-AOC level and SOD activity in the serum of the SEH group were significantly increased (*p* < 0.05). There was no significant difference in MDA content between the SP and SEH groups (*p* > 0.05), but both were lower than that of the CON group (*p* < 0.05).

### 3.4. Serum Immunoglobulin Index

Table 5 shows the effects of SP and SEH on the immunoglobulin levels in the serum of Muscovy ducks. Compared to the CON group, the levels of IgA and IgG in the serum of Muscovy ducks in the SP and SEH groups were significantly increased (*p* < 0.05), but there was no significant difference between the two groups (*p* > 0.05).

### 3.5. Serum Cytokine Indicators

As shown in Table 6, compared to the CON group, the levels of TNF-α, IL-1β, and IL-6 in the serum of the SP and SEH groups were significantly reduced (*p* < 0.05). Notably, the serum levels of TNF-α and IL-6 in the SP group were lower than those in the SEH group (*p* < 0.05). The levels of IL-4 and IL-10 in the serum of the SP group were significantly higher than those in the CON group (*p* < 0.05), while there was no significant difference in IL-4 levels between the SEH and the SP groups (*p* > 0.05). However, the IL-10 levels were lower in the SEH group compared to the SP group (*p* < 0.05).

### 3.6. Intestinal Morphology

As shown in Table 7, compared to the CON group, the VH andV/C of the jejunum in the SP and SEH groups were significantly increased (*p* < 0.05), although no significant differences were observed between the two groups (*p* < 0.05). The CD of the jejunum in the SP and SEH groups was significantly lower than that in the CON group (*p* < 0.05), but again, no significant differences were found between the two groups (*p* < 0.05).

### 3.7. Gut Microbiome

**Figure 1:**
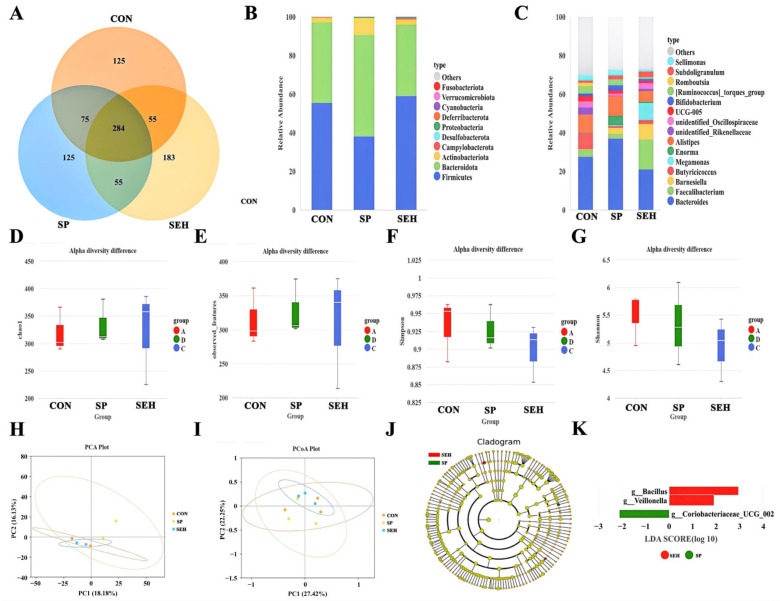
Effects of seaweed polysaccharide and seaweed enzymatic hydrolysis on the cecal microbiome of Muscovy ducks. (**A**) Venn diagram; (**B**) bar chart of relative abundance at the phylum level; (**C**) bar chart of relative abundance at the genus level; (**D**) Chao1 index; (**E**) observed_features index; (**F**) Simpson index; (**G**) Shannon index; (**H**) PCA analysis; (**I**) PCoa analysis; (**J**,**K**) LEfSe analysis. CON group, add 20 mL/kg of water to the basic feed; SP group, add 20 mL/kg of seaweed polysaccharide solution to the basic feed; SEH group, add 20 mL/kg of seaweed enzymatic hydrolysate solution to the basic feed. *n* = 3.

Figure 1 illustrates the impact of SP and SEH on the intestinal microbiome of Muscovy ducks. In the Venn diagram (Figure 1A), the unique ASVs for the CON group, SP group, and SEH group were 125, 183, and 125, respectively. In the bar chart depicting relative abundance at the phylum level (Figure 1B), the abundance proportions of Firmicutes and Bacteroidota exceeded 90% across all three groups. Compared to the CON group, the SP group demonstrated a significant increase in the abundance of Actinobacteriota, while the SEH group exhibited a significant increase in the abundance of Campylobacterota and a notable decrease in the abundance of Deferribacterota. In the bar chart of relative abundance at the phylum level (Figure 1C), compared to the CON group, the abundance of *Barnesiella*, *Enorma*, and *Bifidobacterium* significantly increased in the SP group (*p* < 0.05), while the abundance of *UCG-005* and *Romboutsia* significantly decreased (*p* < 0.05). In the SEH group, the abundance of *Bacteroides*, *Barnesiella*, and *Megamonas* significantly increased (*p* < 0.05), whereas the abundance of *Butyricicoccus*, *UCG-005*, and *Romboutsia* significantly decreased (*p* < 0.05). In the analysis of alpha diversity, there were no significant differences among the Chao1 (Figure 1D), observed features (Figure 1E), Simpson (Figure 1F), and Shannon (Figure 1G) indices between the CON group, SP group, and SEH group (*p* > 0.05). In the analysis of beta diversity, both PCA (Figure 1H) and PCoA (Figure 1I) revealed no significant differences in microbial community composition among the CON group, SP group, and SEH group (*p* > 0.05). The LEfSe analysis (Figure 1J,K) indicated that *g__Bacillus* and *g__Veillonella* were significantly abundant in the SP group (*p* < 0.05), whereas *g__Coriobacteriaceae_UCG_002* was significant in the SEH group (*p* < 0.05).

## 4. Discussion

In the context of pursuing green and sustainable development in the livestock farming industry, the innovation of feed additives has become a key breakthrough for enhancing farming efficiency and product quality [13]. Although the Muscovy duck possesses good growth potential, its growth rate, feed conversion rate, and disease resistance still face bottlenecks in high-density farming environments, necessitating improvements through scientific feed nutritional regulation methods [14]. Seaweeds, as a significant renewable resource in the ocean, have garnered considerable attention in research focused on the development and utilization of their extracts [15]. Seaweed polysaccharides, a type of natural macromolecular carbohydrate derived from seaweeds, demonstrate multiple beneficial effects in animal organisms due to their unique molecular structure, which contains numerous active groups, such as hydroxyl and carboxyl, and exhibits rich biological activity [16,17]. Pradhan et al. reviewed the immunomodulatory, antioxidant, anticancer, and pharmacokinetic activities of polysaccharides extracted from algae, confirming their substantial nutritional value, antioxidant, and anti-inflammatory capabilities, as well as their potential to enhance immune function [18]. Similarly, Shannon et al. analyzed the components of algae as potential modulators of the gut microbiome, positing their potential as prebiotics and their ability to actively regulate the gut microbiota [19]. Another product derived from seaweed is seaweed enzymatic hydrolysate, which is obtained through specific biological enzymatic treatment of seaweed. This technology can decompose the macromolecular substances in seaweed, which are originally difficult for animals to directly absorb, into small molecular active components [9]. This transformation not only retains the various nutritional essences inherent in seaweed but also significantly enhances the absorption and utilization efficiency of these components within the animal body [9]. Currently, there is limited research on SP and SEH in monogastric animals, particularly in ducks. In this experiment, feeding 20 mL/kg of SP and SEH did not significantly affect the ADG of ducks, but it significantly reduced the ADFI and F/G. The reduction in these two indicators typically suggests an improvement in feed conversion efficiency. This positive effect can be explained by the fact that SP and SEH accelerate the absorption and metabolic processes of nutrients in animals, facilitating a more efficient conversion of the energy and nutrients in the feed into animal body tissues, thereby enhancing the economic benefits of farming and resource utilization efficiency.

Serum biochemical indicators, antioxidant indicators, immunoglobulin indicators, and inflammatory factor indicators in blood metrics are important biological markers reflecting the physiological state, metabolic level, and health status of animals [20,21]. ALT and AST are primarily found within hepatocytes; when these cells are damaged, these enzymes are released into the bloodstream in large quantities, leading to increased serum activity [22]. Therefore, they serve as sensitive indicators of liver function. Compared to the CON group, the serum activities of ALT and AST in the SP and SEH groups were significantly reduced, indicating that SP and SEH have a protective effect on the liver. GLU is a core substance in energy metabolism, and its serum level reflects the balance of glucose metabolism in the body, while TG and TCHO are important indicators of lipid metabolism [23]. Zheng et al. studied the effects of polysaccharides from Bangia fusco-purpurea on obesity induced by a high-fat diet (HFD) in C57BL/6 mice, they found that these polysaccharides can enhance energy metabolism, promote lipolysis, increase fatty acid oxidation, and inhibit lipogenesis [24]. The research conducted by Hyun et al. indicates that the L-fucose-rich sulfated polysaccharides derived from edible brown algae can exert potent anti-lipogenic properties by downregulating key regulators of lipogenesis [25]. This result is similar to the regulatory effects of SP on animal lipid metabolism reported in previous studies, suggesting that the supplementation of 20 mL/kg of SP in the diet may exert a positive physiological regulatory effect by improving the glucose and lipid metabolism processes in ducks. The decrease in GLU levels may indicate that SP promotes the utilization or storage of glucose in ducks, maintaining blood sugar levels within a more reasonable range and preventing energy wastage. The reduction in TG and TCHO levels suggests that SP may inhibit fat synthesis or promote its catabolism, thereby reducing fat deposition in the body. The decrease in both LDL-C and HDL-C may be related to the reduction in TCHO.

This experiment also found that SP and SEH improved the antioxidant capacity of the Muscovy duck, specifically reflected in the increased T-AOC and SOD activity in the SEH group, as well as the decreased MDA content in the serum of the SP and SEH groups. Long et al. discovered that the polysaccharide from Gracilaria lemaneiformis can alleviate H_2_O_2_-induced oxidative stress in HepG2 cells through the *Nrf-2/Keap-1* signaling pathway [26]. Matin et al. reviewed the bioactive potential of algae and their derivatives, indicating that algal extracts can inhibit inflammatory signaling pathways such as *NF-κB* and *MAPK*, thereby reducing oxidative damage by activating Nrf2 [27]. Adalbjörnsson et al. found that enzymatic hydrolysis enhances the antioxidant components extracted from seaweeds [28]. This indicates that SP and SEH may enhance the antioxidant capacity of the Muscovy duck by regulating specific signaling pathways or augmenting the activity of antioxidant components.

The immunoglobulin indicators and inflammatory factor indicators are important markers reflecting their immune function and inflammatory status [29]. Immunoglobulins are the core effector molecules of the humoral immunity, and an increase in their levels indicates an enhanced ability of the body to resist pathogen invasion [30]. Inflammatory factors, as signaling molecules of the body’s inflammatory response, when secreted in excess, can trigger chronic inflammation, deplete a large amount of nutrients, and hinder the expression of growth performance [31]. In this experiment, the levels of IgA and IgG in the serum of the SP group and SEH group were significantly increased. Additionally, pro-inflammatory factors such as TNF-α, IL-1β, and IL-6 were significantly reduced, while anti-inflammatory factors IL-4 and IL-10 were significantly elevated. The significant increase in serum levels of IgA and IgG suggests that SP and SEH can enhance the humoral immune response of ducks. IgA, as a core antibody of mucosal immunity, can form a defensive barrier on the mucosal surfaces of the digestive and respiratory tracts, reducing pathogen invasion [32]. IgG, on the other hand, is the most abundant immunoglobulin in body fluids, capable of eliminating pathogens through neutralizing toxins and activating the complement system [33]. The elevation of both antibody levels indicates an enhanced resistance of ducks to diseases, providing an immune guarantee for their healthy growth. The significant decrease in pro-inflammatory factors (TNF-α, IL-1β, IL-6) and the notable increase in anti-inflammatory factors (IL-4, IL-10) reflect that SP and SEH can effectively alleviate excessive inflammatory responses in the body. The excessive secretion of pro-inflammatory factors can lead to tissue damage and the consumption of a large amount of nutrients, while the upregulation of anti-inflammatory factors can inhibit the cascading amplification of inflammation and maintain immune homeostasis [34].

Intestinal morphology is an important indicator reflecting the digestive and absorption functions, intestinal health status, and nutrient utilization efficiency of animals [35]. The structural integrity and morphological characteristics play a crucial role in the growth and development, immune function, and overall health of animals [35]. Currently, there are few reports on the effects of SP and SEH on the intestinal morphology of ducks. However, for monogastric animals, dietary supplementation of seaweed-derived polysaccharides in sows has been shown to enhance the immune response of suckling piglets and improve intestinal morphology [36]. Walsh et al. found that the supplementation of 300 mg/kg of alginate increased both the VH and the V/C in piglets [37]. Additionally, enzymatic hydrolysates of nori can enhance the intestinal mucosal function in obese mice, improve the morphological structure of the small intestine, increase the growth of goblet cells and mucus, elevate the expression level of lysozyme, and stimulate the secretion of sIgA [38]. In this experiment, the supplementation of SP and SEH in the diet significantly increased the VH and the V/C of the jejunum in ducks, while reducing the CD. This indicates that the addition of SP and SEH to the diet can effectively improve the morphological structure of the jejunum in ducks, thereby enhancing their intestinal digestive and absorption functions.

The community structure and functional balance of gut microbiota have a broad and profound impact on the host’s digestion and absorption, immune regulation, metabolic balance, and overall health [39]. Liu et al. found that polysaccharides from *Laminaria Japonica* can enhance the production performance and systemic health of ducks by mediating the gut microbiota [40]. However, there are few reports on the improvement of the gut microbiota structure in Muscovy ducks by SEH. In this experiment, the Alpha and Beta diversity of gut microbiota in ducks supplemented with SP and SEH showed no significant effects. However, in both the SP and SEH groups, a significant increase in the abundance of *Barnesiella* was observed, while the abundances of *UCG-005* and *Romboutsia* significantly decreased. This indicates that SP and SEH may improve the intestinal environment by selectively regulating the core gut microbiota. *Barnesiella* is a beneficial bacterium strongly associated with the synthesis of short-chain fatty acids and anti-inflammatory immune regulation in the gut [41], whereas *UCG-005* and *Romboutsia* are often linked to excessive energy absorption, inflammation risk, and dysbiosis in the intestine [42,43]. LEfSe analysis revealed differences in the dominant bacteria between the SP and SEH groups, with *g__Veillonella* in the SP group primarily involved in the metabolism of carbohydrates and proteins, which helps maintain the normal function and structural integrity of intestinal epithelial cells and regulates intestinal immune function, inhibiting inflammatory responses [44]. Additionally, *g__Coriobacteriaceae_UCG_002* may be related to the metabolic conversion processes of bile acids in the gut. These findings help to explain the different mechanisms by which SP and SEH affect the physiological functions of ducks [45].

## 5. Conclusions

The addition of 20 mL/kg of SP and SEH to the feed significantly improves growth performance and intestinal morphology. At the same time, both substances optimize the intestinal microenvironment by increasing the abundance of *Barnesiella* and decreasing the abundance of *UCG-005* and *Romboutsia*. Furthermore, both SP and SEH enhance metabolic and immune functions in the body, with SEH exhibiting more pronounced antioxidant effects, while SP shows more significant effects in immune regulation. This provides theoretical support for the application of both in the farming of Muscovy ducks.

## Figures and Tables

**Table 1:** Ingredients and nutrient content of experimental diets (%, as-is basis).

Items	1–28 d
Corn	41.50
46% Soybean meal	20.85
Wheat flour	25.00
Rice bran meal	5.50
Extruded Full-fat Soybean	2.50
Limestone	1.75
CaHPO_4_	1.00
NaCl	0.25
98% L-lysine	0.41
98% DL-methionine	0.17
Choline chloride	0.07
Premix ^1^	1.00
Nutrients ^2^	
Digestive energy, MJ/kg	11.97
Crude protein	18.00
Calcium, %	1.05
Phosphorus	0.72
Nonphytate Phosphorus	0.32
Lysine, %	1.10
Methionine, %	0.45
Threonine, %	0.65
Methionine + Cysteine, %	0.78
Tryptophan, %	0.21

^1^ The premix provided the following per kilogram diet: vitamin A 4, 000 IU, vitamin D_3_ 1, 200 IU, vitamin E 10 IU, vitamin K_3_ 8 mg, vitamin B_1_ 8 mg, vitamin B_2_ 3 mg, vitamin B_3_ 20 mg, vitamin B_5_ 40 mg, vitamin B_6_ 1.4 mg, vitamin B_12_ 0.01 mg, biotin 0.05 mg, Cu (as copper sulfate) 8 mg, Fe (as ferrous sulfate) 72 mg, Mn (as manganese sulfate) 100 mg, Zn (as zinc sulfate) 75 mg, Se (as sodium selenite) 0.35 mg, I (as potassium iodide) 0.50 mg. ^2^ Calculated nutrient concentrations.

**Table 2:** Effect of seaweed polysaccharide and seaweed enzymatic hydrolysate on the growth performance of Muscovy ducks.

Items	CON	SP	SEH	SEM	*p*-Value
1 d BW, g	48.82	48.97	49.02	0.281	0.927
28 BW, g	522.67	525.32	525.93	1.337	0.617
ADG, g	18.66	18.75	18.78	0.048	0.614
ADFI, g	33.90 ^a^	31.40 ^b^	31.47 ^b^	0.350	<0.001
F/G	1.82 ^a^	1.67 ^b^	1.68 ^b^	0.021	<0.001

BW, body weight; ADG, average daily gain; ADFI, average daily feed intake; F/G, feed to gain. CON group, add 20 mL/kg of water to the basic feed; SP group, add 20 mL/kg of seaweed polysaccharide solution to the basic feed; SEH group, add 20 mL/kg of seaweed enzymatic hydrolysate solution to the basic feed. Results were presented as the mean and standard error of the mean (SEM), *n* = 4. ^a,b^ In the same row, values with different small letter superscripts mean significant difference (*p* < 0.05).

**Table 3:** Effect of seaweed polysaccharide and seaweed enzymatic hydrolysate on the serum biochemical indicators of Muscovy ducks.

Items	CON	SP	SEH	SEM	*p*-Value
TP, g/L	20.70	19.90	21.20	0.753	0.809
ALB, g/L	8.45	8.38	8.20	0.162	0.841
ALP, U/L	278.35	274.80	275.03	4.899	0.956
ALT, U/L	15.05 ^a^	6.40 ^c^	9.90 ^b^	1.163	<0.001
AST, U/L	27.90 ^a^	12.80 ^c^	19.28 ^b^	2.065	<0.001
GLU, mmol/L	9.63 ^a^	7.39 ^b^	8.30 ^ab^	0.368	0.023
TG, mmol/L	1.48 ^a^	0.88 ^b^	1.18 ^ab^	0.092	0.009
TCHO, mmol/L	2.91 ^a^	1.68 ^b^	2.23 ^ab^	0.204	0.026
LDL-C, mmol/L	1.65 ^a^	0.98 ^b^	1.26 ^ab^	0.122	0.042
HDL-C, mmol/L	1.44 ^a^	0.94 ^b^	1.20 ^ab^	0.092	0.001

TP, total protein; ALB, albumin; ALP, alkaline phosphatase; ALT, alanine aminotransferase; AST, aspartate aminotransferase; GLU, glucose; TG, triglyceride; TCHO, total cholesterol; LDL-C, low-density lipoprotein cholesterol; HDL-C, high-density lipoprotein cholesterol. CON group, add 20 mL/kg of water to the basic feed; SP group, add 20 mL/kg of seaweed polysaccharide solution to the basic feed; SEH group, add 20 mL/kg of seaweed enzymatic hydrolysate solution to the basic feed. Results were presented as the mean and standard error of the mean (SEM), *n* = 4. ^a–c^ In the same row, values with different small letter superscripts mean significant difference (*p* < 0.05).

**Table 4:** Effect of seaweed polysaccharide and seaweed enzymatic hydrolysate on the serum antioxidant capacity of Muscovy ducks.

Items	CON	SP	SEH	SEM	*p*-Value
T-AOC, μmol Trolox/mL	0.27 ^b^	0.30 ^b^	0.38 ^a^	0.015	0.002
SOD, U/mL	18.48 ^b^	20.58 ^b^	24.10 ^a^	0.796	0.001
CAT, μmoL/min/mL	22.09	24.63	24.25	0.884	0.492
GSH-Px, nmol/min/mL	298.20	301.89	292.64	12.309	0.962
MDA, nmol/mL	0.30 ^a^	0.21 ^b^	0.22 ^b^	0.018	0.021

T-AOC, total antioxidant capacity; SOD, superoxide dismutase; CAT, catalase; GSH-Px, glutathione peroxidase; MDA, malondialdehyde. CON group, add 20 mL/kg of water to the basic feed; SP group, add 20 mL/kg of seaweed polysaccharide solution to the basic feed; SEH group, add 20 mL/kg of seaweed enzymatic hydrolysate solution to the basic feed. Results were presented as the mean and standard error of the mean (SEM), *n* = 4. ^a,b^ In the same row, values with different small letter superscripts mean significant difference (*p* < 0.05).

**Table 5:** Effect of seaweed polysaccharide and seaweed enzymatic hydrolysate on the serum immunoglobulin index of Muscovy ducks.

Items	CON	SP	SEH	SEM	*p*-Value
IgA, μg/mL	45.31 ^b^	56.15 ^a^	54.40 ^a^	2.107	<0.001
IgG, μg/mL	321.55 ^b^	475.97 ^a^	450.03 ^a^	10.559	<0.001

IgA, immunoglobulin A; IgG, immunoglobulin G. CON group, add 20 mL/kg of water to the basic feed; SP group, add 20 mL/kg of seaweed polysaccharide solution to the basic feed; SEH group, add 20 mL/kg of seaweed enzymatic hydrolysate solution to the basic feed. Results were presented as the mean and standard error of the mean (SEM), *n* = 4. ^a,b^ In the same row, values with different small letter superscripts mean significant difference (*p* < 0.05).

**Table 6:** Effect of seaweed polysaccharide and seaweed enzymatic hydrolysate on the serum cytokine indicators of Muscovy ducks.

Items	CON	SP	SEH	SEM	*p*-Value
TNF-α, pg/mL	16.37 ^a^	11.15 ^c^	13.75 ^b^	0.668	<0.001
IL-1β, pg/mL	54.26 ^a^	36.48 ^b^	41.50 ^b^	2.526	<0.001
IL-6, pg/mL	7.03 ^a^	3.77 ^c^	4.87 ^b^	0.423	<0.001
TGF-β1, ng/mL	39.49	38.53	41.61	2.507	0.071
IL-4, pg/mL	20.59 ^b^	29.17 ^a^	30.25 ^a^	1.542	0.004
IL-10, pg/mL	11.12 ^b^	14.39 ^a^	12.00 ^b^	0.541	0.017

TNF-α, tumor necrosis factor-α; IL-1β, interleukin-1β; IL-6, interleukin-6; TGF-β1, transforming growth factor-β1; IL-4, interleukin-4; IL-10, interleukin-10. CON group, add 20 mL/kg of water to the basic feed; SP group, add 20 mL/kg of seaweed polysaccharide solution to the basic feed; SEH group, add 20 mL/kg of seaweed enzymatic hydrolysate solution to the basic feed. Results were presented as the mean and standard error of the mean (SEM), *n* = 4. ^a–c^ In the same row, values with different small letter superscripts mean significant difference (*p* < 0.05).

**Table 7:** Effect of seaweed polysaccharide and seaweed enzymatic hydrolysate on the intestinal morphology of Muscovy ducks.

Items	CON	SP	SEH	SEM	*p*-Value
VH, μm	660.79 ^b^	753.32 ^a^	766.65 ^a^	12.361	<0.001
CD, μm	221.26 ^a^	200.13 ^b^	198.48 ^b^	5.521	<0.001
V/C	2.99 ^b^	3.76 ^a^	3.86 ^a^	0.177	<0.001

VH, Villus height; CD, Crypt depth; V/C, Villus height/Crypt depth. CON group, add 20 mL/kg of water to the basic feed; SP group, add 20 mL/kg of seaweed polysaccharide solution to the basic feed; SEH group, add 20 mL/kg of seaweed enzymatic hydrolysate solution to the basic feed. Results were presented as the mean and standard error of the mean (SEM), *n* = 4. ^a,b^ In the same row, values with different small letter superscripts mean significant difference (*p* < 0.05).

## Data Availability

The raw data supporting the conclusions of this article will be made available by the authors, without undue reservation. The raw data of the cecal microbiota of Muscovy (PRJNA1302354) were uploaded to NCBI.

## References

[1] Liu M., Luan H., Qiu W., Zhang Y., Feng W., Xu W., Wang F., Xuan H., Song P. (2025). Antibiotic alternatives in livestock feeding. Sci. Total. Environ..

[2] Lin R., Li H., Lai L., Yang F., Qiu J., Lin W., Bao X., Pan C., Lin W., Jiang X. (2024). Analysis of genetic structure and identification of important genes associated with muscle growth in Fujian Muscovy duck. Poult. Sci..

[3] Li Z., Zhou H., Liu W., Wu H., Li C., Lin F., Yan L., Huang C. (2024). Beneficial effects of duck-derived lactic acid bacteria on growth performance and meat quality through modulation of gut histomorphology and intestinal microflora in Muscovy ducks. Poult. Sci..

[4] Li Z., Li C., Lin F., Yan L., Wu H., Zhou H., Guo Q., Lin B., Xie B., Xu Y. (2024). Duck compound probiotics fermented diet alters the growth performance by shaping the gut morphology, microbiota and metabolism. Poult. Sci..

[5] Ivarsson E., Wall H., Wistedt A., Cervin G., Pavia H., Wattrang E. (2025). Effects of algal supplementation on broiler chicken growth performance, gut development, blood leukocyte counts and antibody levels. Animal.

[6] Guo X., Chen J., Yang J., He Q., Luo B., Lu Y., Zou T., Wang Z., You J. (2021). Seaweed polysaccharide mitigates intestinal barrier dysfunction induced by enterotoxigenic *Escherichia coli* through NF-κB pathway suppression in porcine intestinal epithelial cells. J. Anim. Physiol. Anim. Nutr..

[7] Michalak I., Tiwari R., Dhawan M., Alagawany M., Farag M.R., Sharun K., Emran T.B., Dhama K. (2022). Antioxidant effects of seaweeds and their active compounds on animal health and production—A review. Vet. Q..

[8] Terriente-Palacios C., Rubiño S., Hortós M., Peteiro C., Castellari M. (2022). Taurine, homotaurine, GABA and hydrophobic amino acids content influences “in vitro” antioxidant and SIRT1 modulation activities of enzymatic protein hydrolysates from algae. Sci. Rep..

[9] Cermeño M., Kleekayai T., Amigo-Benavent M., Harnedy-Rothwell P., FitzGerald R.J. (2020). Current knowledge on the extraction, purification, identification, and validation of bioactive peptides from seaweed. Electrophoresis.

[10] Ribeiro D.M., Martins C.F., Costa M., Coelho D., Pestana J., Alfaia C., Lordelo M., de Almeida A.M., Freire J.P.B., Prates J.A.M. (2021). Quality Traits and Nutritional Value of Pork and Poultry Meat from Animals Fed with Seaweeds. Foods.

[11] Guo Y., Zhao Z.-H., Pan Z.-Y., An L.-L., Balasubramanian B., Liu W.-C. (2020). New insights into the role of dietary marine-derived polysaccharides on productive performance, egg quality, antioxidant capacity, and jejunal morphology in late-phase laying hens. Poult. Sci..

[12] Corino C., Di Giancamillo A., Modina S.C., Rossi R. (2021). Prebiotic Effects of Seaweed Polysaccharides in Pigs. Animals.

[13] Fonseca A., Kenney S., Van Syoc E., Bierly S., Dini-Andreote F., Silverman J., Boney J., Ganda E. (2024). Investigating antibiotic free feed additives for growth promotion in poultry: Effects on performance and microbiota. Poult. Sci..

[14] Zhu Z., Huang B., Sun N., Yu X., Du Z., Li A., Huang C. (2024). Variations in gut microbiota composition and reproductive hormone levels between laying and broody Muscovy ducks. Poult. Sci..

[15] Véliz K., Toledo P., Araya M., Gómez M.F., Villalobos V., Tala F. (2023). Chemical composition and heavy metal content of Chilean seaweeds: Potential applications of seaweed meal as food and feed ingredients. Food Chem..

[16] Otero P., Carpena M., Garcia-Oliveira P., Echave J., Soria-Lopez A., Garcia-Perez P., Fraga-Corral M., Cao H., Nie S., Xiao J. (2023). Seaweed polysaccharides: Emerging extraction technologies, chemical modifications and bioactive properties. Crit. Rev. Food Sci. Nutr..

[17] Liyanage N.M., Nagahawatta D.P., Jayawardena T.U., Jeon Y.-J. (2023). The Role of Seaweed Polysaccharides in Gastrointestinal Health: Protective Effect against Inflammatory Bowel Disease. Life.

[18] Pradhan B., Bhuyan P.P., Ki J.-S. (2023). Immunomodulatory, Antioxidant, Anticancer, and Pharmacokinetic Activity of Ulvan, a Seaweed-Derived Sulfated Polysaccharide: An Updated Comprehensive Review. Mar. Drugs.

[19] Shannon E., Conlon M., Hayes M. (2021). Seaweed Components as Potential Modulators of the Gut Microbiota. Mar. Drugs.

[20] Yang W., Lang X., Song D., Xu H., Zhang C., Guo L., Chen X. (2024). Comparative analysis of reproductive hormones, serum biochemical indexes and ovarian metabolites in Muscovy breeder duck at different laying stages. Poult. Sci..

[21] Zhang Y.N., Wang S., Huang X.B., Li K.C., Chen W., Ruan D., Xia W.C., Wang S.L., Abouelezz K.F.M., Zheng C.T. (2020). Estimation of dietary manganese requirement for laying duck breeders: Effects on productive and reproductive performance, egg quality, tibial characteristics, and serum biochemical and antioxidant indices. Poult. Sci..

[22] Feng T., Li S., Wang P., Zhu D., Xu Z., Wang L., Li A., Kulyar M.F., Shen Y. (2024). Hepatoprotective effects of Radix Bupleuri extract on aflatoxin B1-induced liver injury in ducks. Ecotoxicol. Environ. Saf..

[23] Zhang B., Li M., Zhou G., Gu X., Xie L., Zhao M., Xu Q., Tan G., Zhang N. (2023). ZnO-NPs alleviate aflatoxin B1-induced hepatoxicity in ducklings by promoting hepatic metallothionein expression. Ecotoxicol. Environ. Saf..

[24] Zheng M., Chao X., Zheng Y., Hong T., Wu W., Zhu Y., Ni H., Jiang Z. (2024). A polysaccharide from edible red seaweed Bangia fusco-purpurea prevents obesity in high-fat diet-induced C57BL/6 mice. Int. J. Biol. Macromol..

[25] Hyun J., Lee H.-G., Je J.-G., Choi Y.-S., Song K.-M., Kim T.-K., Ryu B., Kang M.-C., Jeon Y.-J. (2024). L-Fucose-Rich Sulfated Glycans from Edible Brown Seaweed: A Promising Functional Food for Obesity and Energy Expenditure Improvement. Int. J. Mol. Sci..

[26] Long X., Hu X., Pan C., Xiang H., Chen S., Qi B., Liu S., Yang X. (2022). Antioxidant Activity of *Gracilaria lemaneiformis* Polysaccharide Degradation Based on Nrf-2/Keap-1 Signaling Pathway in HepG2 Cells with Oxidative Stress Induced by H_2_O_2_. Mar. Drugs.

[27] Matin M., Koszarska M., Atanasov A.G., Król-Szmajda K., Jóźwik A., Stelmasiak A., Hejna M. (2024). Bioactive Potential of Algae and Algae-Derived Compounds: Focus on Anti-Inflammatory, Antimicrobial, and Antioxidant Effects. Molecules.

[28] Adalbjörnsson B.V., Jónsdóttir R. (2015). Enzyme-Enhanced Extraction of Antioxidant Ingredients from Algae. Methods Mol. Biol..

[29] Wu S., Wang L., Cui B., Wen X., Jiang Z., Hu S. (2023). Effects of Vitamin A on Growth Performance, Antioxidants, Gut Inflammation, and Microbes in Weaned Piglets. Antioxidants.

[30] Volf J., Kaspers B., Schusser B., Crhanova M., Karasova D., Stepanova H., Babak V., Rychlik I. (2024). Immunoglobulin secretion influences the composition of chicken caecal microbiota. Sci. Rep..

[31] Kober A.K.M.H., Saha S., Ayyash M., Namai F., Nishiyama K., Yoda K., Villena J., Kitazawa H. (2024). Insights into the Anti-Adipogenic and Anti-Inflammatory Potentialities of Probiotics against Obesity. Nutrients.

[32] Chen Y., Li X., Yang M., Jia C., He Z., Zhou S., Ruan P., Wang Y., Tang C., Pan W. (2024). Time-restricted eating reveals a “younger” immune system and reshapes the intestinal microbiome in human. Redox Biol..

[33] Quan S., Huang J., Chen G., Zhang A., Yang Y., Wu Z. (2024). Genistein Promotes M2 Macrophage Polarization via Aryl Hydrocarbon Receptor and Alleviates Intestinal Inflammation in Broilers with Necrotic Enteritis. Int. J. Mol. Sci..

[34] Li D., Meng K., Liu G., Wen Z., Han Y., Liu W., Xu X., Song L., Cai H., Yang P. (2025). Lactiplantibacillus plantarum FRT4 protects against fatty liver hemorrhage syndrome: Regulating gut microbiota and FoxO/TLR-4/NF-κB signaling pathway in laying hens. Microbiome.

[35] Aldis R.E., Muhlisin M., Zuprizal Z., Sasongko H., Hanim C., Anas M.A. (2024). Black soldier fly larvae meal supplementation in a low protein diet reduced performance, but improved nitrogen efficiency and intestinal morphology of duck. Anim. Biosci..

[36] Heim G., O’Doherty J.V., O’Shea C.J., Doyle D.N., Egan A.M., Thornton K., Sweeney T. (2015). Maternal supplementation of seaweed-derived polysaccharides improves intestinal health and immune status of suckling piglets. J. Nutr. Sci..

[37] Walsh A.M., Sweeney T., O’SHea C.J., Doyle D.N., O’DOherty J.V. (2012). Effects of supplementing dietary laminarin and fucoidan on intestinal morphology and the immune gene expression in the weaned pig. J. Anim. Sci..

[38] Li C., Cheng X., Cao W., Wang Y., Xue C., Tang Q. (2022). Enzymatic hydrolysate of porphyra enhances the intestinal mucosal functions in obese mice. J. Food Biochem..

[39] Zhu Z., Liao L., Su J., Liu Z., Pan S., Huang Y., Wu Y. (2022). Interactions of Muscovy duck reovirus, gut microbiota, and host innate immunity: Transcriptome and gut microbiota analysis. Vet. Microbiol..

[40] Liu A., Kim E., Cui J., Li J., Lee Y., Zhang G. (2023). *Laminaria Japonica* Polysaccharide Improved the Productivities and Systemic Health of Ducks by Mediating the Gut Microbiota and Metabolome. J. Agric. Food Chem..

[41] Qin S., Zhu Y., Tian G., Jensen M.B., Zhang K., Ding X., Bai S., Wang J., Xuan Y., Zeng Q. (2025). Dietary resistant starch protects against post-antibiotic intestinal damage by restoring microbial homeostasis and preserving intestinal barrier function in meat duck. Poult. Sci..

[42] Hao Y., Ji Z., Shen Z., Wu Y., Zhang B., Tang J., Hou S., Xie M. (2021). Effects of Total Dietary Fiber on Cecal Microbial Community and Intestinal Morphology of Growing White Pekin Duck. Front. Microbiol..

[43] Shi X., Huang M., Song J., Zeng L., Liang Q., Qu Y., Li J., Xu G., Zheng J. (2022). Effects of different duck rearing systems on egg flavor and quality and microbial diversity. Poult. Sci..

[44] Yang H., Jo H., Kim S.H., Yun C.-S., Park S.-H., Park D.-S. (2024). *Veillonella faecalis* sp. nov., a propionic acid-producing bacterium isolated from the faeces of an infant. Antonie Van Leeuwenhoek.

[45] Feng J., Teng Z., Yang Y., Liu J., Chen S. (2024). Effects of semaglutide on gut microbiota, cognitive function and inflammation in obese mice. PeerJ.

